# Weight Growth Velocity and Neurodevelopmental Outcomes in Extremely Low Birth Weight Infants

**DOI:** 10.1371/journal.pone.0139014

**Published:** 2015-09-24

**Authors:** Hidehiko Maruyama, Naohiro Yonemoto, Yumi Kono, Satoshi Kusuda, Masanori Fujimura, Neonatal Research Network of Japan

**Affiliations:** 1 Department of Pediatrics, Kochi Health Sciences Center, Kochi, Kochi, Japan; 2 Department of Neuropsychopharmacology, National Center of Neurology and Psychiatry, Kodaira, Tokyo, Japan; 3 Department of Pediatrics, Jichi Medical University, Shimotsuke, Tochigi, Japan; 4 Department of Neonatology, Maternal and Perinatal Center, Tokyo Women's Medical University, Tokyo, Japan; 5 Department of Neonatology, Osaka Medical Center and Research Institute for Maternal and Child Health, Izumi, Osaka, Japan; Hôpital Robert Debré, FRANCE

## Abstract

**Introduction:**

This study aimed to assess whether weight growth velocity (WGV) predicts neurodevelopmental outcomes in extremely low birth weight infants (ELBWIs).

**Methods:**

Subjects were infants who weighed 501–1000 g at birth and were included in the cohort of the Neonatal Research Network of Japan (2003–2007). Patel’s exponential model (EM) method was used to calculate WGV between birth and discharge. Assessment of predictions of death or neurodevelopmental impairment (NDI) was performed at 3 years of age based on the WGV score, which was categorized by per one increase in WGV. Multivariate logistic regression analysis was used to calculate adjusted odds ratios and their 95% confidence intervals (95%CI).

**Results:**

In the 2961 ELBWIs assessed, the median WGV was 10.5 g/kg/day (interquartile, 9.4–11.9). With the categorical approach, the adjusted odds ratios for death or NDI with WGV scores of 6 and 7 were 2.41 (95%CI, 1.60–3.62) and 1.81 (95%CI, 1.18–2.75), respectively, relative to the reference WGV score of 10. WGV scores ≥8 did not predict death or NDI.

**Conclusions:**

WGV scores <8 were significant predictors suggesting that values of WGV during hospitalization in a NICU are associated with neurodevelopmental outcomes. Further investigations is necessary to determine whether additional nutritional support may improve low WGV in ELBWIs.

## Introduction

Postnatal weight gain in extremely low birth weight infants (ELBWIs) is a critical issue for neurodevelopment outcomes [1]. Respiratory, circulatory and nutritional support contribute to the appropriate and gradual weight increase in ELBWIs [2]. However, postnatal weight in ELBWIs can dramatically change postpartum depending on the conditions and acute or chronic morbidities after birth. An early and aggressive nutritional regimen, including parenteral and enteral nutrition, is effective in improving growth [3]. Previous studies have reported that adequate weight gain resulted in better neurodevelopmental outcomes compared with poor weight gain [1, 4, 5]. Weight growth velocity (WGV) is an appropriate index to use when assessing postnatal weight gain. However, no consensus exists in the field regarding measurement methods for WGV.

Patel et al. recently developed the exponential model (EM) for WGV measurement [6, 7]. However, no studies have reported on the predictive value of WGV as determined by the EM method for neurodevelopmental outcomes of ELBWIs. This study aimed to assess whether WGV, calculated using the EM method, can predict neurodevelopmental outcomes at the age of 3 years in ELBWIs.

## Materials and Methods

### Study design and participants

Data on infants born between 2003 and 2007 were obtained from the prospective cohort of the Neonatal Research Network of Japan (NRNJ). All participating centers of the network are designated tertiary NICUs by the local government as well as the national government. The database contains information on the morbidity and mortality of very low birth weight infants (VLBWIs) with birth weight (BW) ≤1500 g. Considering the nation-wide population of VLBWIs born in Japan during the study period, more than 50% of VLBW infants in Japan were in the registry [8]. Our selection criteria were surviving infants with BWs between 501–1000 g at discharge, at institutions with follow-up rates of over 60% at the age of 3 years. Infants who died before discharge, had major congenital malformations, those who were gestational age <22 weeks and had a length of stay <30 days were excluded. Length of stay was used as an exclusion criterion because growth velocity during the first month could not be calculated. The inclusion and exclusion criteria used herein are the same as those described by Patel et al [6, 7].

### Weight growth velocity (WGV)

WGV was calculated using the EM method reported by Patel et al [6, 7]. WGV is commonly used as a growth measure in VLBW infants, as it summarizes infant weight gain over a specific time interval, smoothing the variability that is inherent in daily weight measures. However, calculating growth velocity (GV) from daily weight measures by averaging these values over the desired time interval is extremely labor-intensive. The EM method allows accurate estimation of postnatal GV in ELBW infants during hospitalization in a NICU without the influence of length of stay, BW, or the presence of chronic lung disease. Comparisons of the EM method with four other methods (2-point BW model, 2-point average weight model, linear BW model and linear average weight model) have confirmed the superiority of the EM method in terms of accuracy [6, 7], with mean magnitudes of error of <0.1% regardless of starting point or time interval, and no errors exceeding 5% of the accurate standard GV for any of the infants analyzed.

The EM method is easy to use from the clinical perspective as it only requires information on weight and age at two time points. WGV (g/kg/day) was calculated with the EM method as follows:

WGV = (1000 x ln (Wn/W1))/(Dn−D1)

wherein, W1 is BW, Wn is weight at discharge, D1 is 0 and Dn is day at discharge. Patel’s EM assumes that growth occurs at a fraction of the previous weight (otherwise known as “first-order kinetics,” with the unit of g/kg/day), and average growth velocity is estimated based on the fact that growth in biological systems is generally nonlinear, often following an exponential pattern.

The WGV score, which was categorized by per one increase in WGV, was used in the categorical analysis. A WGV score of 6 represented WGV <7, a WGV score of 7 represented 7≤ WGV <8, and so on up to a WGV score of 13. A WGV score of 14 represents WGV ≥14.

### Confounding factors

The following were adjusted for as confounding factors: maternal age, primiparous, maternal diabetes, pregnancy-induced hypertension, clinical chorioamnionitis, preterm rupture of membrane (PROM), antenatal corticosteroid, cesarean section, sex, gestational age (GA), BW, Apgar score (AS) (1 and 5 min), respiratory distress syndrome (RDS), chronic lung disease (CLD), patent ductus arteriosus (PDA), necrotizing enterocolitis (NEC) and intraventricular hemorrhage (IVH) [1, 5, 9, 10]. Clinical chorioamnionitis was diagnosed based on the clinical findings, such as maternal fever, leukocytosis and local pain during pregnancy, labor or delivery [11]. Antenatal corticosteroid use was defined as administration of at least 1 dose of corticosteroid to the mother at any time before delivery to accelerate fetal lung maturity. GA was determined by the best estimate based on early prenatal ultrasound examination, the last menstrual period and the physical examination of infants at birth. Small for gestational age (SGA) was defined as a BW less than the 10th percentile of the standard BW for gestational age published by the Japan Pediatric Society [12]. RDS was defined as RDS diagnosed by clinical and radiographic findings. CLD was defined as the oxygen requirement at corrected 36 weeks. PDA was diagnosed clinically or echocardiographically. NEC was defined using Bell classification stage II or greater [13]. The grade of IVH was diagnosed with cranial echography according to the classification of Papile, with grades III and IV corresponding to severe IVH [14].

### Outcomes

The primary outcome was death or neurodevelopmental impairment (NDI) at the age of 3 years (i.e., 36–42 months). NDI was defined as any one of the following: cerebral palsy, unilateral or bilateral blindness, severe hearing impairment or developmental delay.

NDI was determined with a comprehensive follow-up protocol to evaluate signs and symptoms of cerebral palsy (CP), sensory abnormality and cognitive function [15]. Cerebral palsy was defined as a non-progressive central nervous system disorder characterized by abnormal muscle tone in at least one extremity and abnormal control of movement and posture [16]. Visual impairment included unilateral or bilateral blindness based on a diagnosis by ophthalmologists. Severe hearing impairment was defined based on the need of a hearing aid.

Developmental delay was defined as a developmental quotient (DQ) <70 measured using the Kyoto Scale of Psychological Development (KSPD) test or judged by physicians for infants who did not undergo the test [15, 17]. The assessors of the KSPD test at each center were blinded to the perinatal details but were informed of gestational age. The physicians were not always blinded to perinatal and neonatal morbidities and interventions.

The KSPD test was administered by psychologists working in each participating center. The latest version of the KSPD was standardized in 2001 for 2677 Japanese children, and the mean and one standard deviation of DQ were 100.6 and 13.4, respectively [18]. Developmental function of infants whose DQ were not measured by the KSPD was evaluated by physicians based on whether the infants were able to say meaningful words, say their own name or age and distinguish between circles with diameters of 4 cm and 6 cm at each center [17, 19].

### Statistical Analysis

Clinical characteristics of the subjects were stratified based on the following WGV distributions: WGV <9, 9≤ WGV <12 and WGV ≥12. Cutoff values of WGV 9 and 12 were used because the 25th and 75th percentiles of WGVs were 9.4 and 11.9, respectively. Univariate logistic regression analyses were performed to assess the effects of WGV score on death and NDI. Odds ratios (ORs) and their 95% confidence intervals (95% CIs) were calculated using simple descriptive analysis. Multivariate logistic regression analyses were performed to adjust for confounding factors. These factors included: primiparous, pregnancy-induced hypertension, clinical chorioamnionitis, antenatal corticosteroid, male sex, GA, BW, AS at 5 min <7, RDS, CLD, PDA, NEC and IVH, which have been known to affect the neurodevelopmental outcome in VLBWIs [1, 20, 21]. Adjusted odds ratios (AORs) and their 95% CIs were also calculated. We calculated that the planned sample size should give over 80% power to detect the difference in all variables after adjustment, with an OR of 1.5.

Categorical scales were used to analyze WGV scores because the relationship between WGV and outcomes may not be linear (i.e., the categorical scales help to understand the non-linear relationship). Validity was confirmed using descriptive approaches. In the categorical analysis, WGV scores were set as categorical variables, with the reference WGV score of 10. This value was chosen because the mean WGV was 10.4 and median WGV was 10.5. In the categorical analysis, ORs and AORs are based on WGV scores versus the reference score. Subgroup analyses were also performed for extremely preterm infants: GA <28 weeks vs. GA ≥28 weeks [22, 23], BW <750 g vs. BW ≥750 g[15, 24] and SGA vs. non SGA [25]. Consistent with Patel’s indication that EM should only be used for infants with a length of stay <160 days [7], sensitivity analysis was performed by excluding infants with a length of stay ≥160 days.

In order to avoid selection bias from missing data for WGV and outcomes, we performed multiple imputations (MI) with the assumption that data were missing at random [26]. MI is a Monte Carlo technique in which the missing values are replaced by stochastic and multiple simulated data by modeling with assumption of the missing mechanism. The MI model was based on year of birth, center, plurality, antenatal steroid, sex, out-born, birth weight, GA, CLD at 36 weeks, IVH III-IV, PVL, sepsis, NEC and intestinal perforation, which were found to correlate to the adverse outcome in the previous study [27]. This model allowed us to draw valid inferences from the repeated imputed data in the analysis of incomplete data. Statistical analyses were performed using JMP 10.0.2 (SAS Institute, Inc., Cary, NC, USA) and SAS 9.2 (SAS Institute, Inc., Cary, NC, USA).

The study protocol was approved by the Ethics Review Committee of Jichi Medical University and is available to the public on the Internet [28]. Parents provided written informed consent for their child’s participation.

## Results

**Fig 1:**
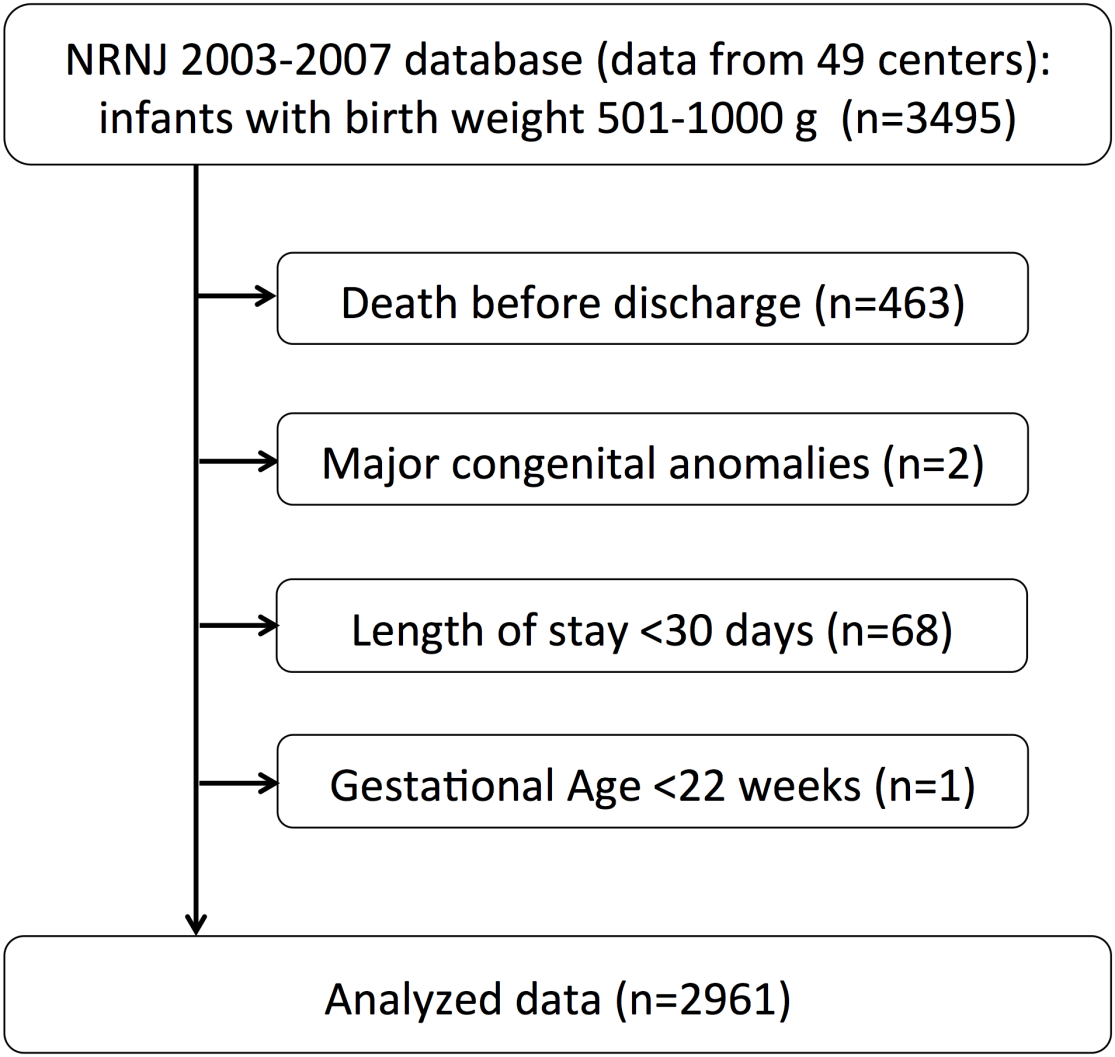
Study flowchart. Analyzed data (n = 2961) were obtained from the Neonatal Research Network of Japan 2003–2007 database.

**Fig 2:**
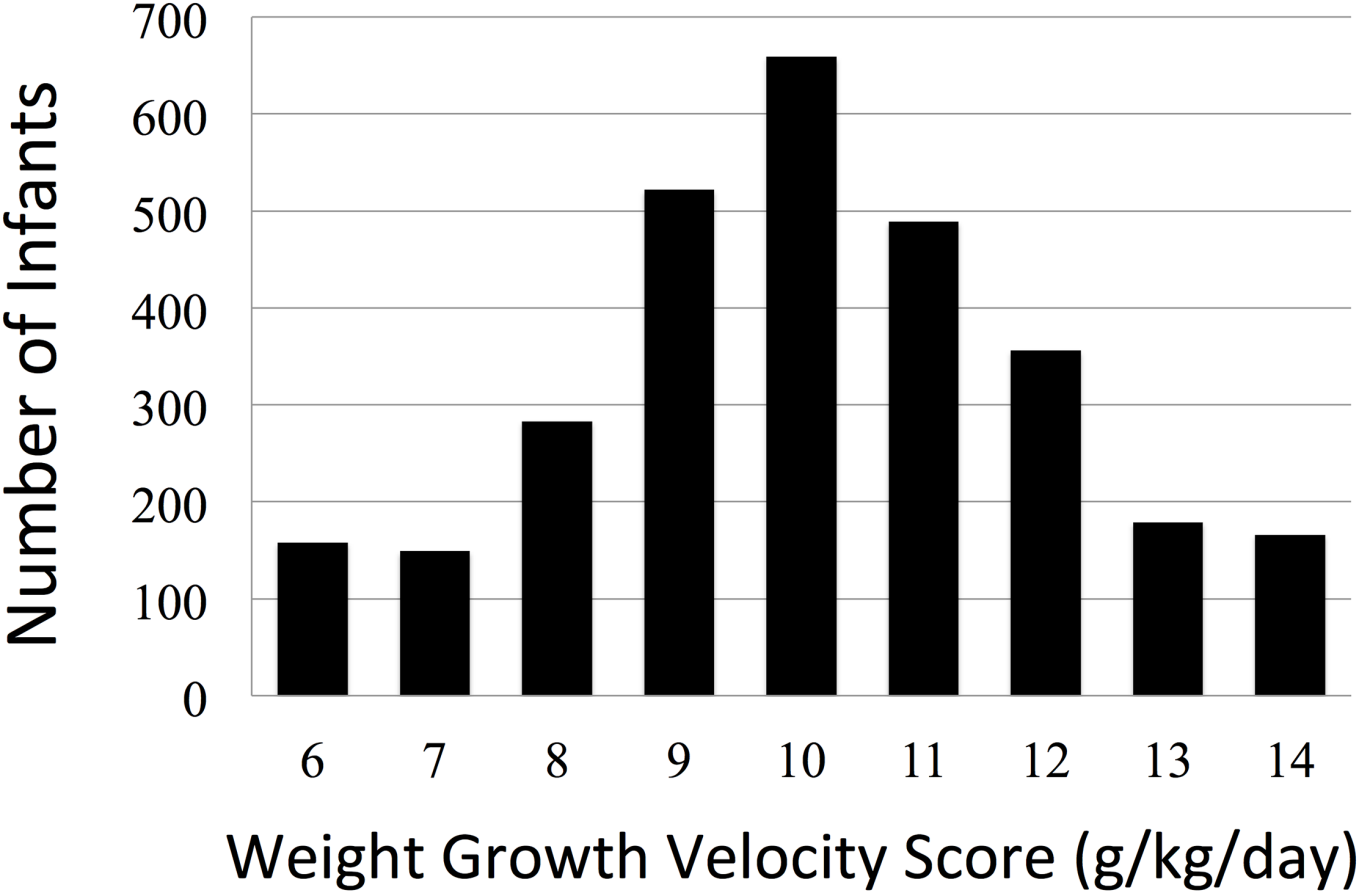
Histogram of weight growth velocity (WGV) scores.

In the NRNJ 2003–2007 database, which includes data from 49 centers, there were 3495 eligible infants with BWs between 501 and 1000 g. After excluding 534 cases as shown in Fig 1, we analyzed 2961 cases including 1189 cases that were lost to follow-up. The outcomes of patients we were able to follow included no NDI (1223), NDI (531) and death (18). Of the 1754 NDI and no-NDI cases, 1468 had undergone the KSPD test. NDI cases included 214 cases with cerebral palsy, 38 with visual impairment, 25 with hearing impairment and 350 with developmental delay (294 with DQ <70 and 56 judged by physicians). 89 infants had more than one on these impairments. There were 358 infants with missing WGV data and 113 overlap cases. The missing values of WGV and neurodevelopmental outcomes were imputed by the MI method. Clinical characteristics of the data-imputed infants (n = 1189 + 358–113 = 1434) were early GA, small BW and many morbidities such as RDS, CLD, PDA, NEC and IVH. After MI, there were 18 deaths post-discharge (0.6%) and 533 NDI cases (18.0%) among the eligible cases. Clinical characteristics of the subjects divided into nine groups by WGV scores are shown in Table 1. The WGV score distribution is shown in Fig 2. The mean WGV was 10.6 g/kg/day (SD 2.5), and the median WGV was 10.5 (interquartile, 9.4–11.9; min-max, 0–44.4). The distribution before MI was similar to this result, with the mean (SD) WGV of 10.8 (2.5) g/kg/day (n = 1527). The mean WGV score of the study population was 10. There were some differences in clinical characteristics between infants with the KSPD test and those without. The clinical characteristics of infants without the KSPD test included early GA and some morbidities, such as RDS and NEC.

**Table 1:** Clinical characteristics by weight growth velocity (WGV) score.

WGV score		6 (n = 158)	7 (n = 149)	8 (n = 283)	9 (n = 522)	10 (n = 659)	11 (n = 489)	12 (n = 356)	13 (n = 179)	14 (n = 166)	Total (n = 2961)
Maternal age*	Mean(SD)	30.4(5.2)	30.0(5.1)	30.8(5.2)	30.5(5.4)	30.7(5.3)	30.8(5.2)	31.4(5.3)	31.4(5.0)	32.1(4.8)	30.8(5.2)
Primiparous	n(%)	87(55.1)	81(54.5)	138(48.8)	281(53.8)	394(59.8)	296(60.5)	226(63.5)	107(59.8)	119(71.7)	1729(58.4)
Diabetes*	n(%)	1(0.6)	3(2.0)	4(1.4)	6(1.2)	7(1.1)	7(1.4)	6(1.7)	1(0.6)	1(0.6)	36(1.2)
Hypertension	n(%)	11(7.0)	13(8.7)	30(10.6)	58(11.1)	78(11.8)	96(19.6)	79(22.2)	47(26.3)	59(35.5)	471(15.9)
Chorioamnionitis*	n(%)	42(26.8)	45(30.2)	70(24.8)	130(25.2)	125(19.1)	90(18.5)	57(16.0)	28(15.7)	14(8.5)	601(20.4)
PROM*	n(%)	58(36.7)	60(40.3)	94(33.2)	176(33.8)	206(31.3)	133(27.2)	90(25.3)	43(24.0)	23(13.9)	883(29.8)
Antenatal Corticosteroid*	n(%)	57(36.1)	61(40.9)	116(41.0)	235(45.1)	289(43.9)	201(41.2)	175(49.2)	85(47.5)	80(48.5)	1299(43.9)
Cesarean section	n(%)	119(75.3)	114(76.5)	211(74.6)	374(71.7)	477(72.4)	377(77.1)	294(82.6)	150(83.8)	149(89.8)	2265(76.5)
Male*	n(%)	90(57.0)	92(61.7)	141(49.8)	277(53.1)	320(48.7)	217(44.5)	173(48.7)	90(50.3)	92(55.4)	1492(50.5)
Gestational age	Mean(SD)	26.4(2.4)	25.8(1.9)	26.2(1.9)	26.0(1.9)	26.4(1.8)	27.0(2.2)	27.6(2.4)	28.0(2.7)	29.4(2.6)	26.8(2.3)
Birth weight	Mean(SD)	752(139)	752(141)	784(135)	772(137)	781(137)	779(140)	787(134)	797(144)	809(139)	780(138)
SGA	n(%)	49(31.0)	34(22.8)	61(21.6)	113(21.7)	188(28.5)	206(42.1)	186(52.3)	106(59.2)	140(84.3)	1083(36.6)
AS 1 min <7*	n(%)	139(88.5)	123(83.7)	237(84.0)	393(76.5)	481(74.6)	350(72.2)	244(69.1)	109(61.6)	98(59.4)	2174(74.3)
AS 5 min <7*	n(%)	74(48.1)	69(46.9)	112(40.0)	178(36.7)	197(32.9)	147(31.2)	84(24.2)	42(23.7)	17(10.4)	920(32.6)
RDS	n(%)	115(72.8)	115(77.2)	215(76.0)	363(69.5)	472(71.6)	336(68.7)	221(62.1)	101(56.4)	61(36.8)	1999(67.5)
CLD	n(%)	96(60.8)	109(73.2)	179(63.3)	331(63.4)	360(54.6)	247(50.5)	163(45.8)	68(38.0)	39(23.5)	788(26.9)
PDA	n(%)	87(55.1)	93(62.4)	145(51.2)	287(55.0)	296(44.9)	194(39.7)	133(37.4)	57(31.8)	28(16.9)	1320(44.6)
NEC	n(%)	12(7.6)	4(2.7)	9(3.2)	8(1.5)	8(1.2)	2(0.4)	0(0)	0(0)	0(0)	43(1.5)
IVH	n(%)	28(17.7)	29(19.5)	60(21.2)	106(20.3)	107(16.2)	82(16.8)	46(12.9)	23(12.9)	14(8.4)	495(16.7)
Severe IVH	n(%)	8(5.1)	8(5.4)	19(6.7)	38(7.3)	32(4.9)	21(4.3)	15(4.2)	2(1.1)	4(2.4)	147(5.0)
Day at discharge	Mean(SD)	253(164)	180(51.8)	148(37.8)	138(51.1)	127(51.5)	111(26.1)	102(21.6)	95(20.9)	78(19.1)	130(65.7)

Continuous variables are expressed as mean (SD). Categorical variables are expressed as incidence (%).

* indicates that data for some infants were missing.

The number of missing values for the WGV scores 6 to 14 and total, respectively, are as follows:

Maternal age: n = 12, 1, 11, 20, 34, 49, 34, 12, 17, 190; Diabetes: n = 0, 0, 0, 1, 0, 0, 0, 0, 0, 1; Chorioamnionitis: n = 1, 0, 1, 6, 4, 3, 0, 1, 1, 17; PROM: n = 0, 0, 0, 1, 1, 0, 0, 0, 0, 2; Antenatal corticosteroid: n = 0, 0, 0, 1, 0, 1, 0, 0, 1, 3; Male sex: n = 0, 0, 0, 0, 2, 1, 1, 0, 0, 4; AS 1 min <7: n = 1, 2, 1, 8, 14, 4, 3, 2, 1, 36; AS 5 min <7: n = 4, 2, 3, 37, 61, 18, 9, 2, 2, 138.

WGV; weight growth velocity, SD; standard deviation, PROM; preterm rupture of membrane, SGA; small for gestational age, AS; Apgar score, RDS; respiratory distress syndrome, CLD; chronic lung disease, PDA; patent ductus arteriosus, NEC; necrotizing enterocolitis, IVH; intraventricular hemorrhage.

**Fig 3:**
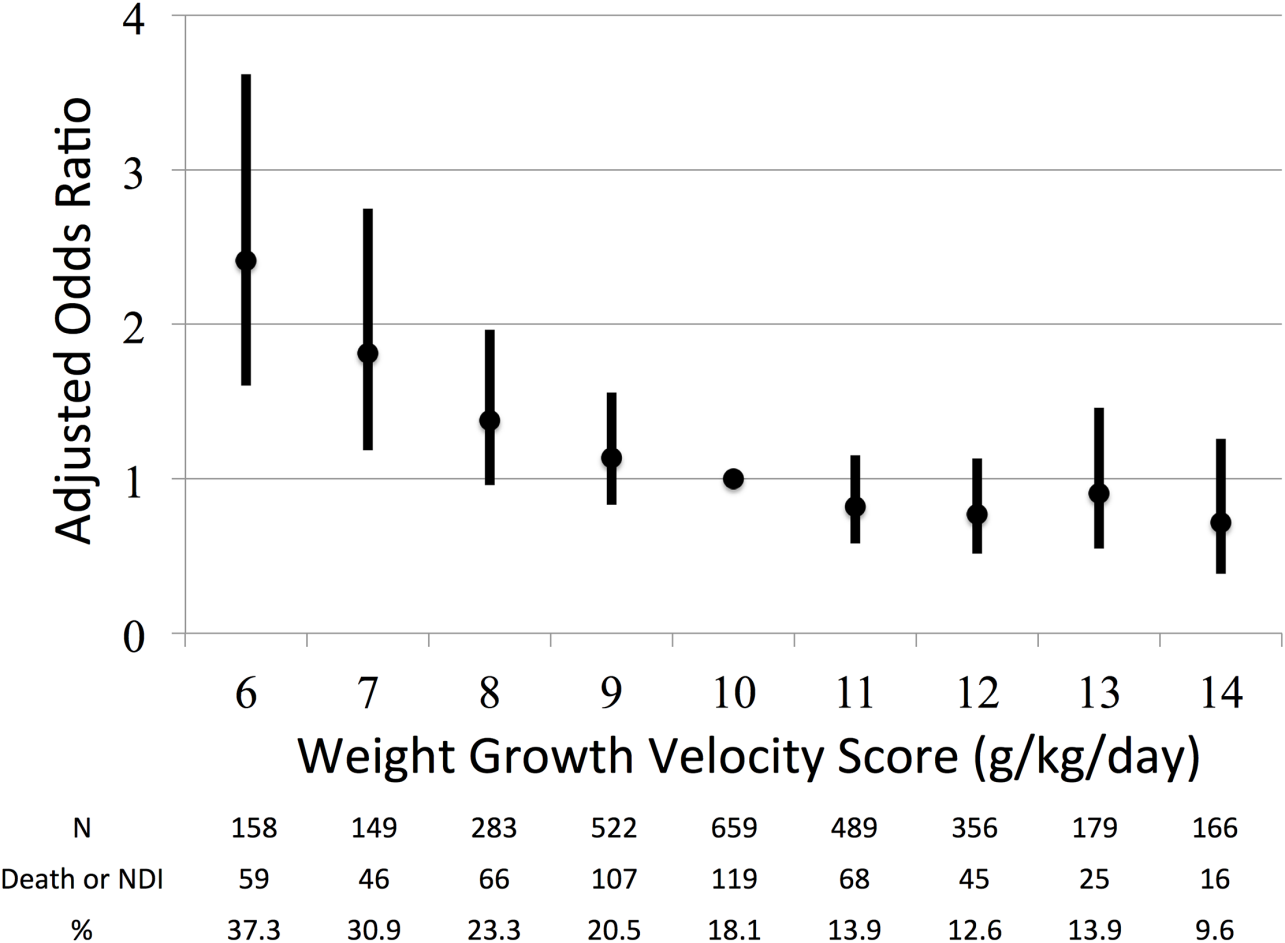
Relationship between weight growth velocity (WGV) scores 6–14 and their adjusted odds ratios (AORs) with 95% confidence intervals (CIs). WGV scores 6 and 7 predicted death or NDI at 3 years of age.

With the categorical approach, only WGV scores 6 (AOR, 2.41; 95%CI, 1.60–3.62) and 7 (AOR, 1.81; 95%CI, 1.18–2.75) predicted death or NDI at 3 years of age (Table 2 and Fig 3). The AUC was 0.67. Subgroup analyses for the primary outcome were similar to the main analysis, with the exception of GA ≥28 weeks. AOR of the infants in the group GA ≥28 weeks was not significantly related to the WGV score, except that AOR in the WGV score 6 was 3.57 (95%CI, 1.32–9.69). In the sensitivity analysis after excluding 495 infants with extremely long stays in the NICU, AORs of WGV scores 6 and 7 were 0.58 (95%CI, 0.17–1.54) and 1.87 (95%CI, 0.93–3.58), respectively.

**Table 2:** Logistic regression analysis for death or NDI in categorical analysis (n = 2961).

WGV score	n	OR	95%CI	p-value	AOR	95%CI	p-value
6	158	2.70	1.85–3.94	<.0001	2.41	1.60–3.62	<.0001
7	149	2.03	1.35–3.01	0.0008	1.81	1.18–2.75	0.006
8	283	1.38	0.98–1.93	0.065	1.38	0.96–1.97	0.081
9	522	1.17	0.87–1.56	0.291	1.14	0.83–1.56	0.423
10	659	1	-	-	1	-	-
11	489	0.73	0.53–1.01	0.058	0.82	0.58–1.15	0.254
12	356	0.66	0.45–0.94	0.023	0.77	0.52–1.13	0.184
13	179	0.74	0.45–1.16	0.189	0.91	0.55–1.46	0.696
14	166	0.48	0.27–0.82	0.006	0.72	0.39–1.26	0.253

The reference value was the WGV score 10.

WGV; weight growth velocity, OR; odds ratio, AOR; adjusted odds ratio, CI; confidence interval.

## Discussion

This is the first study to report on the predictive ability of WGV calculated by the EM method with adjusted confounding factors using multivariate analysis. Using this method, we found that WGV scores 6 and 7, relative to the reference median WGV score of 10, predicted neurodevelopmental outcomes in ELBWIs. Our findings suggest that low WGV scores (i.e., ≤7) are associated with unfavorable outcomes, and ELBWIs with WGV scores ≤7 require careful follow-up and support.

Previous studies have used the best versus worst group approach. For example, Ehrenkranz et al. compared neurodevelopmental outcomes between maximum and minimum weight gain groups [1]. In this study, we analyzed data using a categorical approach, which we believe to be more appropriate, given that comparisons are made relative to the median, which is the normal and representative value in a population.

AORs for middle and high WGV scores did not significantly predict outcomes in this study. This result is comparable to that reported by Pylipow et al., in which the effects of excessive weight gain were less pronounced with better neurodevelopmental outcomes than those of suboptimal weight gain [10]. This may explain why high WGV scores did not predict neurodevelopmental outcomes in the present study.

The AUC in the categorical analysis for the primary outcome was 0.67, which is not high. Such an AUC would have limited predictive ability over all infants. Although we attempted to identify specific populations with high AUCs in the subgroup analysis, the results were inconclusive.

The characteristics of infants and the standard criteria for discharge may differ by country and medical care system. Our data were collected from over 40 centers in Japan, and it should be noted that the medical care system and universal health coverage at these centers were homogeneous. In Patel’s study, the study population consisted of ELBWIs from one hospital with the following parameters: a mean GA of 26.2 weeks, BW of 785.0 g, length of the NICU stay of 77.6 days (range; 34–175), weight at discharge of 2087.5g and WGV of 12.675 g/kg/day [7]. Although GA and BW of our cases were similar to those cases, the length of NICU stay and body weight at discharge were longer and heavier. In our sensitivity analysis, the results changed after excluding infants with extremely long stays in the NICU. According to Patel et al., caution is required when applying the EM to infants with long stays [7]. The mean WGV in the present study was 10.6 g/kg/day, which is slightly lower than 12.7 g/kg/day reported by Patel et al. The differences between our and their results could be attributed to clinical strategies for weight gain and timing to discharge.

We recommend that a baby should be reevaluated if WGV is less than 8, particularly with regard to his/her nutritional state. However, it is difficult to distinguish clearly between good and bad WGVs in the current status, as there might be differences in the distribution of WGV scores for each population. In fact, the mean WGV scores varied between our study and Patel’s study. As studies that report WGV scores based on the EM method are limited, future investigation is warranted.

### Study limitations

This study has several limitations worth noting. First, the KSPD test evaluates neurodevelopment, and thus our results may be difficult to compare with studies that used other measures, such as the Bayley Scales of Infant Development III [27]. Second, our data included outcomes that were judged by pediatricians without the KSPD test. As previously discussed [27], infants at 22–23 weeks of age are more likely to be examined by pediatricians, and are more likely to have other conditions such as blindness and CP. Thus, this might have introduced some bias. Third, there were missing data for some infants who were not followed up. To address this issue, we used MI to draw inferences with the assumption that data were missing at random. However, if this assumption was inappropriate, our estimation could be deemed invalid. Fourth, we had no data about the feeding. A recent study showed that breastfeeding lead to better neurodevelopment in spite of suboptimal initial weight gain in very preterm infants [29]. In order to generalize our results, it will be necessary to conduct a study with large population-based data. We are currently working to identify interventions for managing WGV in order to achieve and support satisfactory neurodevelopmental outcomes.

## Conclusion

WGV scores <8 were significant predictors of death and NDI at the age of 3 years in ELBWIs, supporting the clinical usefulness of WGV scores. Our findings also suggested an association between WGV values during hospitalization in a NICU and neurodevelopmental outcomes. Further investigation is necessary to determine whether additional nutritional support may improve low WGV in ELBWIs.

## Abbreviations

AOR: adjusted odds ratio
AS: Apgar score
BW: birth weight
CI: confidence interval
CLD: chronic lung disease
DQ: developmental quotient
ELBWI: extremely low birth weight infant
EM: exponential model
GA: gestational age
IVH: intraventricular hemorrhage
KSPD: Kyoto Scale of Psychological Development
NDI: neurodevelopmental impairment
NEC: necrotizing enterocolitis
OR: odds ratio
PDA: patent ductus arteriosus
PROM: preterm rupture of membrane
RDS: respiratory distress syndrome
SD: standard deviation
SGA: small for gestational age
VLBWI: very low birth weight infants
WGV: weight growth velocity
